# Alcohol Drinking Pattern and Risk of Head and Neck Cancer: A Nationwide Cohort Study

**DOI:** 10.3390/ijerph182111204

**Published:** 2021-10-25

**Authors:** Hye Yeon Koo, Kyungdo Han, Dong Wook Shin, Jung Eun Yoo, Mi Hee Cho, Keun Hye Jeon, Dahye Kim, Sangduk Hong, Jae Kwan Jun

**Affiliations:** 1 Health Promotion Center, CHA Bundang Medical Center, Seongnam 13496, Korea; tare4645@chamc.co.kr; 2 Department of Statistics and Actuarial Science, Soongsil University, Seoul 06978, Korea; hkd@ssu.ac.kr; 3 Department of Family Medicine/Supportive Care Center, Samsung Medical Center, Sungkyunkwan University School of Medicine, Seoul 06351, Korea; 4 Department of Digital Health, SAIHST, Sungkyunkwan University, Seoul 06355, Korea; 5 Center for Clinical Epidemiology, SAIHST, Sungkyunkwan University, Seoul 06355, Korea; 6 Department of Family Medicine, Healthcare System Gangnam Center, Seoul National University Hospital, Seoul 06236, Korea; 83259@snuh.org; 7 Samsung C&T Medical Clinic, Kangbuk Samsung Hospital, Sungkyunkwan University School of Medicine, Seoul 05288, Korea; mh76.cho@samsung.com; 8 Department of Family Medicine, CHA Gumi Medical Center, CHA University, Gumi 39295, Korea; kh1228@chamc.co.kr; 9 Department of Medical Statistics, The Catholic University of Korea, Seoul 07345, Korea; dhkim373@daewoong.co.kr; 10 Department of Otorhinolaryngology, Samsung Medical Center, Sungkyunkwan University School of Medicine, Seoul 06351, Korea; sangduk.hong@samsung.com; 11 National Cancer Control Institute, National Cancer Center, Goyang 10408, Korea; jkjun@ncc.re.kr; 12 Graduate School of Cancer Science and Policy, National Cancer Center, Goyang 10408, Korea

**Keywords:** head and neck neoplasms, alcohol drinking, drinking behavior, risk factors, cohort studies

## Abstract

Alcohol consumption is a major risk factor for head and neck cancer (HNC), yet little data exist examining drinking patterns and HNC risk. In this population-based, retrospective cohort study, 11,737,467 subjects were recruited from the Korean National Health Insurance Service database. The risks of overall HNC and HNC subtypes according to average alcohol consumption, drinking frequency, and daily amount were examined using Cox proportional hazard models. Over the median follow-up of 6.4 years, 15,832 HNC cases were identified. HNC risk linearly increased with drinking frequency (*p*-trend < 0.01; adjusted hazard ratio [aHR] 1.55, 95% confidence interval [CI] 1.45–1.67 in subjects who drank 7 days/week). HNC risk also increased according to daily amount of alcohol consumption (*p*-trend < 0.01), but plateaued from 5–7 units/occasion (aHR 1.25, 95% CI 1.19–1.31) to >14 units/occasion (aHR 1.26, 95% CI 1.13–1.40). When stratified by average alcohol consumption, drinking frequency, but not daily amount, showed a linear relationship with HNC risk in moderate and heavy drinkers. When comparing the HNC subtypes, similar tendencies were observed in cancers of the oral cavity, pharynx, and larynx, but not in the salivary gland. In conclusion, drinking frequency is a stronger risk factor for HNC, especially for cancer of the oral cavity, pharynx, and larynx, than the daily amount of alcohol consumption.

## 1. Introduction

As global cancer incidence and cancer death are rapidly growing, identifying modifiable cancer risk factors is becoming more important [1,2]. There were about 470,000 global death cases from head and neck cancer (HNC)–including cancers of the lip, oral cavity, salivary gland, pharynx, and larynx–in 2020 [1]. Approximately 900,000 patients in the world and 5000 patients in Korea are newly diagnosed with HNC annually [1]. Along with cigarette smoking, alcohol consumption is a major independent risk factor for HNC, and reduced exposure to these factors is expected to prevent more than half of the squamous cell carcinomas of the head and neck [3,4].

Previous studies of HNC risk have mostly focused on the average alcohol consumption level [5,6,7,8]. These studies, including meta-analyses [7,8], have almost consistently shown that average alcohol intake (g/day) is associated with HNC risk. However, recent reports on several types of cancers, such as breast and prostate cancer, have suggested that drinking patterns, including binge drinking and frequency of drinking, might also affect the carcinogenic effect of alcohol [9,10]. In relation to HNC, little data exists on drinking patterns and cancer risk. One study based on two large prospective cohorts observed that the risk of alcohol-related cancers, including cancers of oral cavity, pharynx, larynx, and others, increased with the frequency of drinking in men even when adjusted for total alcohol intake (*p* for trend = 0.03) [11]. In another prospective study, the association between the risk of upper aerogastric tract cancers and drinking frequency was examined; however, the daily amount of drinking was not considered [12]. Drinking 4–7 times per week was associated with an increased cancer risk compared to non-drinkers (relative risk [RR] 3.9, 95% confidence interval [CI] 2.1–7.1) [12]. However, previous cohort studies were based on a limited number of cancer cases (417 [11] and 49 HNC cases [12], respectively), and the risk of various HNC subtypes according to drinking pattern were not examined. In a study by the INHANCE consortium, joint effects of drinking intensity (number of drinks per day) and duration (years) on risks of HNC subtypes were examined [13]. This study has included a relatively large number of HNC cases (4100), yet the analysis was based on case-control results. Risks of all HNC subtypes increased according to alcohol intensity without threshold, whereas risks were not considerably modified by duration except for that of the oropharynx. However, the effect of drinking frequency was not investigated. Another case-control report, which included 240 cancer cases, has observed a linear relationship between drinking frequency and overall upper aerodigestive tract cancers (*p* for trend = 0.01) [14], whereas no association was observed between oral cancer subtype (*n* = 187) and drinking frequency in a study based on the same data [15]. There are few meta-analyses or systematic reviews on drinking frequency and overall HNC risk, but one meta-analysis of cohort studies has examined the association between nasopharyngeal cancer risk and drinking frequency [16]. Frequent drinking (≥7 times per week) was related to an increase in the risk of nasopharyngeal cancer (pooled odds ratio [OR] 1.29, 95% CI 1.05–1.53) in this analysis, whereas infrequent drinking (<7 times per week) showed the contrasting result (pooled OR 0.77, 95% CI 0.60–0.94).

In our study, we aimed to investigate the association between alcohol drinking patterns and the risk of overall HNC or HNC subtypes using a large population-based cohort. The effects of two components of drinking patterns -drinking frequency and daily amount- were compared using stratified analyses by average alcohol consumption.

## 2. Materials and Methods

### 2.1. Study Population and Data Source

The Korean National Health Insurance Service (NHIS) database was used for this study. The National Health Insurance Service is a mandatory social insurance service managed by the Korean government that provides universal health coverage to nearly the whole population of South Korea [17]. NHIS also provides all subscribers over 40 years old with a national health screening every two years; this program includes assessment of health-related behaviors, such as smoking and drinking, as well as anthropometric and laboratory tests. Therefore, the NHIS database contains almost all medical information, including clinical diagnoses, medical procedures, prescription records, and sociodemographic factors, of its beneficiaries.

From this database, 12,724,396 subjects who were aged over 40 years and who received national health screening in 2009–2010 were initially included. Subjects who were diagnosed with any cancer before the health screening (*n* = 303,424), who had any cancer within one year from the health screening (*n* = 124,472), or who were deceased within one year from the health screening (*n* = 16,182) were excluded. Subjects who had any missing data (*n* = 542,851) were also excluded. The final study population comprised 11,737,467 subjects.

Study subjects were followed from one year after the national health screening date to the date of incident HNC, death, or the last day of the study period (31 December 2017), whichever came first. The median follow-up time was 6.4 years, after a one-year lag period.

### 2.2. Ethical Approval

This study was approved by the Institutional Review Board of Samsung Medical Center (IRB File No. SMC 2019-02-059). The requirement for written informed consent from patients was waived because the data were anonymized under confidentiality guidelines.

### 2.3. Exposure to Alcohol

Data on alcohol consumption was collected from the self-reported questionnaires used for the national health examination in 2009–2010. This questionnaire includes questions about the average drinking frequency (number of days per week) during the last year and the daily amount (standard units per occasion) of drinking, regardless of the type of alcoholic beverage.

Based on this data, we calculated the pure alcohol intake per occasion first. Standard drink size seems to vary among countries, possibly due to cultural differences. [18,19]. One standard unit is commonly defined as 8 g of pure alcohol in Korea, probably resulting from Korean drinking custom [20]. The majority of alcohol consumed in Korea is beer and Soju (a traditional Korean alcohol beverage), and the amounts of pure alcohol contained in a usual size of cup used for beer (220 mL) and Soju (50 mL) in Korea are estimated to be about 8 g [20,21]. Hence, we assumed that one standard unit (one cup of each alcohol beverage type) described in a questionnaire of NHIS contains 8 g of pure alcohol.

The average weekly alcohol consumption (g/week) was calculated by multiplying the pure alcohol intake per occasion and drinking frequency. Since even a light-to-moderate amount of drinking might increase the risk of cancer [11], the average alcohol consumption level was classified in detail as follows: (1) non-drinker (0 g/week); (2) mild drinker (0–105 g/week); (3) moderate drinker (105–210 g/week); and (4) heavy drinker (≥ 210 g/week) [16].

### 2.4. Covariates

Information on potential confounders, including age, sex, income level, smoking status, physical activity, body mass index (BMI), and comorbidities, were collected from the NHIS claims database and the national health screening database. The level of income was categorized into quartiles. Smoking status was categorized into never smoker, ex-smoker, and current smoker; ex-smoker and current smoker were each further categorized into <20 pack-years of smoking history and ≥20 pack-years of history. Physical activity level was categorized into non-regular and regular exercise; the regular exercise group was defined as performing either >30 min of moderate physical activity at least five times/week or >20 min of strenuous physical activity at least three times/week. BMI was calculated by kg/m^2^. Baseline comorbidities, including hypertension, diabetes mellitus, and dyslipidemia, were defined based on a combination of health examination records, disease diagnosis codes, and prescription records.

### 2.5. Study Outcomes

Diagnosis of new HNC was the endpoint of this study. Incidence of HNC was identified using claims records with given diagnosis codes, according to the International Classification of Disease, 10th Revision (ICD-10): cancers of the oral cavity (C00–C06), salivary gland (C07, C08), pharynx (C09–C13), larynx (C32), and other sites in the head and neck (C14, C30, C31) were defined as HNC. In addition, a diagnosis of cancer was also identified based on the receipt of a special co-payment reduction program for critical illness. In Korea, cancer patients who possess a medical certificate of diagnosis can apply for the special co-payment reduction program. The enrollees of this program pay only 5% of medical expenses for cancer work-up and cancer treatment, whereas other patients pay usually 20–30% of expenses for common diseases. Therefore, nearly all cancer patients are registered in this program in Korea, which makes our information on HNC diagnosis extremely reliable.

### 2.6. Statistical Analysis

Descriptive statistics were used to examine the baseline characteristics of the study population according to average alcohol consumption level. A Student’s *t*-test was performed to compare continuous variables, and a chi-squared test was performed to compare categorical variables.

Incidence rates of overall HNC and HNC subtypes were calculated as the sum of new cases during the follow-up period divided by the sum of the person-years (per 100,000). To evaluate the effect of each independent variable, including average alcohol consumption, drinking frequency, and daily amount, on the risk of developing overall HNC or one of the HNC subtypes, Cox proportional hazard regression analyses were conducted. For all analyses, non-drinkers were used as the reference group. We also adjusted for all of the following covariates in gradual modeling: (1) Model 1 was adjusted for age and sex as a basic analysis; (2) Model 2 was additionally adjusted for variables that generally show associations with HNC or were commonly considered in previous studies, including income, smoking status, physical activity level, BMI and diabetes mellitus [5,14,22,23]; (3) Model 3 was additionally adjusted for common comorbidities which were associated with HNC in a few studies, including hypertension and dyslipidemia [24,25].

The risks of overall HNC and HNC subtypes were stratified by average alcohol consumption level. The impacts of drinking frequency (days per week) and daily amount (standard units per occasion) were examined.

All analyses were performed using SAS version 9.4 (SAS Institute Inc., Cary, NC, USA), and the two-sided significance levels were set at *p* < 0.05.

## 3. Results

### 3.1. Baseline Demographics

Table 1 shows the baseline characteristics of the study population. About 40% of subjects (*n* = 4,727,135) were drinkers. When compared with non-drinkers, drinkers were younger, male, in lower socioeconomic status, and had a smoking history (ex- or current smoker). The proportion of ever-smokers increased with increasing average alcohol consumption level (*p* < 0.0001).

### 3.2. Risk of HNC According to Drinking Status

**Figure 1:**
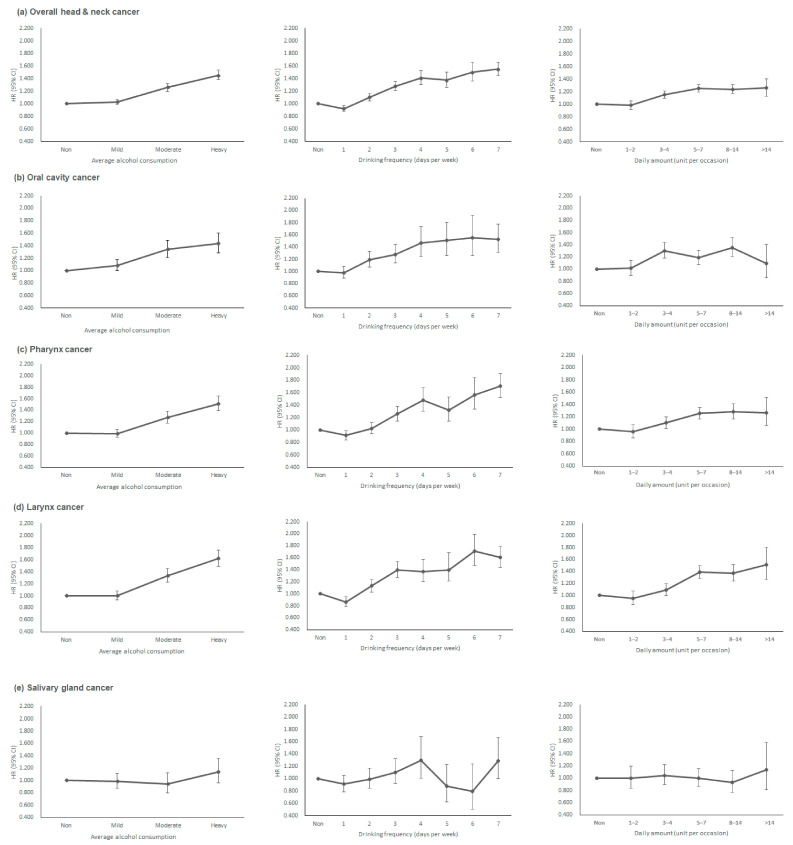
Risk of head & neck cancer according to alcohol drinking status. HR, hazard ratio; CI, confidence interval. HRs are adjusted for age, sex, income, smoking status, physical activity, body mass index, diabetes mellitus, hypertension and dyslipidemia. (**a**) overall head & neck cancer, (**b**) oral cavity cancer, (**c**) pharynx cancer, (**d**) larynx cancer, (**e**) salivary gland cancer.

15,832 HNC cases were identified during the follow-up period. The risk of developing HNC according to average alcohol consumption, drinking frequency (days per week), and daily amount (standard units per occasion) are presented in Table 2 and Figure 1. When comparing the effects of average alcohol consumption on HNC risk, mild drinkers showed no significant difference in HNC risk compared to non-drinkers, but moderate and heavy drinkers had a higher risk of HNC (adjusted hazard ratio [aHR] 1.26, 95% CI 1.20–1.32 and aHR 1.46, 95% CI 1.38–1.53, respectively, in Model 3). As for drinking frequency, HNC risk increased almost linearly in a dose-response manner (*p*-trend < 0.01): the risk began to significantly increase in subjects who drank 2 days/week (aHR 1.10, 95% CI 1.05–1.16) compared to non-drinkers and was highest in subjects who drank 7 days/week (aHR 1.55, 95% CI 1.45–1.67). HNC risk was also increased according to the daily amount per occasion (*p*-trend < 0.01): it continuously increased in subjects who drank ≤5–7 standard units per occasion when compared to non-drinkers, and then plateaued afterwards (aHR 1.26, 95% CI 1.13–1.40 in subjects who drank >14 units/occasion). Regarding the adjustment model, the addition of comorbidities of hypertension and dyslipidemia (Model 3) did not produce a substantially different result from that of Model 2.

The results of analyses on HNC subtypes and drinking status are presented in Table 3 and Figure 1. Cancers of the oral cavity and pharynx cancers showed similar results to those associated with overall HNC risk; risks of these cancers increased almost linearly as average alcohol consumption and drinking frequency increased (aHR 1.44, 95% CI 1.29–1.60, and aHR 1.51, 95% CI 1.39–1.64, respectively, in heavy drinkers; aHR 1.53, 95% CI 1.31–1.78, and aHR 1.71, 95% CI 1.53–1.91, respectively, in subjects who drank 7 days/week). Risks of these cancers were also increased in subjects who drank more than 3–4 standard units per occasion (aHR 1.30, 95% CI 1.18–1.45, and aHR 1.10, 95% CI 1.01–1.20, respectively, in subjects who drank 3–4 units/occasion), but this increased risk was less prominent with higher daily amounts of alcohol. In the case of larynx cancer, cancer risk increased almost linearly according to average alcohol consumption, drinking frequency, and daily amount (aHR 1.62, 95% CI 1.49–1.76 in heavy drinkers; aHR 1.61, 95% CI 1.44–1.79 in subjects who drank 7 days/week; aHR 1.51, 95% CI 1.27–1.80 in subjects who drank > 14 units/day). No definite tendency was observed for the risk of salivary gland cancer according to drinking status.

### 3.3. Risk of HNC and Drinking Pattern: Drinking Frequency vs. Daily Amount

**Figure 2:**
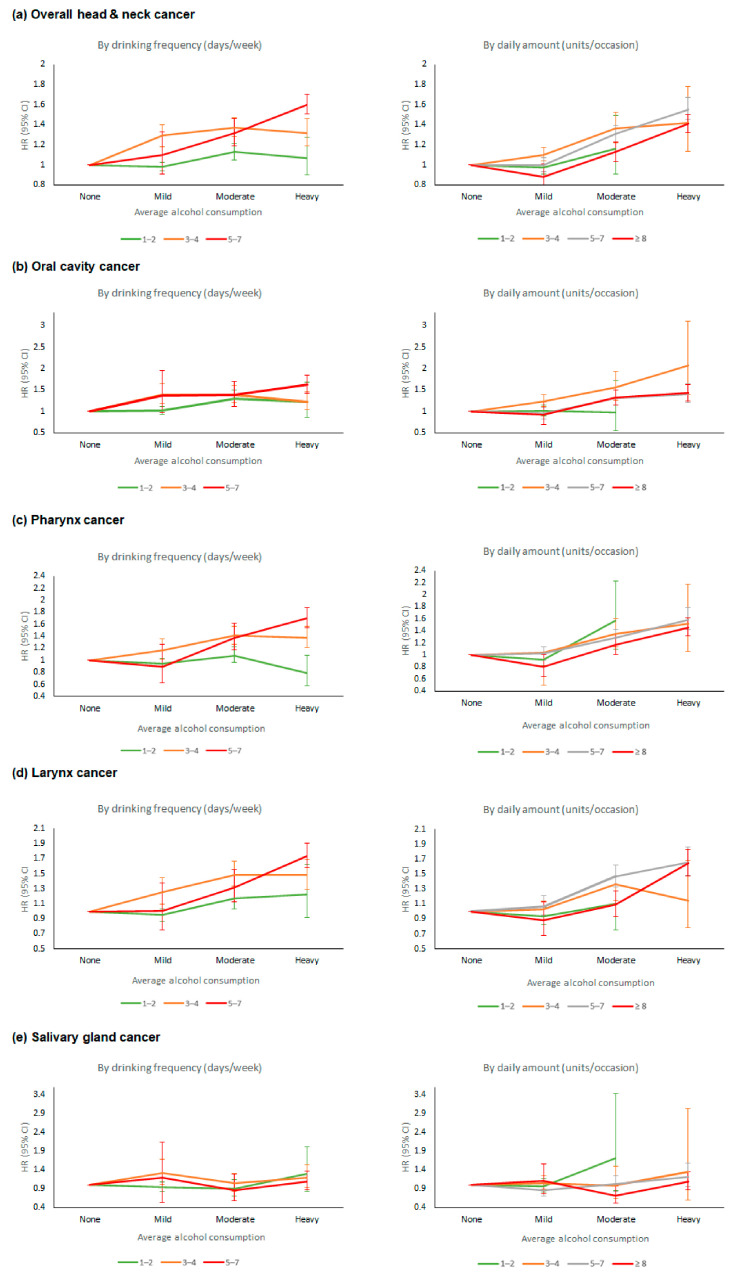
Risk of head & neck cancer stratified by average alcohol consumption: Impact of drinking frequency versus daily amount. (**a**) overall head & neck cancer, (**b**) oral cavity cancer, (**c**) pharynx cancer, (**d**) larynx cancer, (**e**) salivary gland cancer. HR, hazard ratio; CI, confidence interval. HRs are adjusted for age, sex, income, smoking status, physical activity, body mass index, and diabetes mellitus.

Supplementary Table S1 and Figure 2 show the risk of overall HNC stratified by average alcohol consumption. In the mild drinker group, no definite trend was observed according to either drinking frequency or daily amount. For drinking frequency, the risk of HNC was highest among subjects who drank 3–4 days/week (aHR 1.29, 95% CI 1.18–1.40 in Model 2) in mild drinkers. As for the daily amount, HNC risk was highest in subjects who drank 3–4 units/occasion (aHR 1.10, 95% CI 1.04–1.17 in Model 2).

Moderate drinkers showed a nearly linear increase in HNC risk according to drinking frequency (aHR 1.32, 95% CI 1.19–1.46 in subjects who drank 5–7 days/week), whereas HNC risk decreased with increasing daily amount among subjects who drank at least 3–4 units/occasion.

In heavy drinkers, HNC risk linearly increased according to drinking frequency (aHR 1.60, 95% CI 1.51–1.70 in subjects who drank 5–7 days/week), whereas no definite difference in HNC risk was observed according to the daily amount.

When comparing the associations between drinking patterns and risks of developing one of the HNC subtypes, similar tendencies were observed in cancers of the oral cavity, pharynx, and larynx, but not salivary gland cancers (Supplementary Table S2 and Figure 2).

## 4. Discussion

In this large-scale, nationwide, cohort study, average alcohol consumption was associated with an increased risk of overall HNC. The frequency of drinking was almost linearly associated with the risk of overall HNC. The daily amount of drinking also showed a positive association with overall HNC risk, but the level of association was weaker than that observed with drinking frequency. When comparing HNC subtypes, cancers of the oral cavity, pharynx, and larynx showed similar patterns as those of overall HNC, but salivary gland cancer showed no definite tendency according to drinking status.

We found that average alcohol consumption was positively associated with HNC risk, which is consistent with previous research. Numerous studies have reported that drinking is an independent risk factor of HNC and that average alcohol consumption level is strongly associated with HNC risk in a dose-response manner [5,7,14,26,27,28]. The carcinogenic effect of alcohol is speculated to mainly result from acetaldehyde (AA), which is the metabolite generated from ethanol [29,30]. Suggested mechanisms of AA-related cancer development include interruption of the copying of DNA during cell division, interference in DNA repair, and induction of mutations by forming DNA adducts [29,31]. Recent studies have reported that ethanol metabolism can occur in the oral cavity independently from the liver [32,33], which is in line with findings that AA has been observed in saliva in higher levels than those found in blood, and salivary AA concentration increases dose-dependently according to alcohol consumption [29,34]. Hence, salivary AA is thought to contribute to direct carcinogenesis in the oral cavity and pharynx [29,35].

When comparing HNC subtypes, associations of similar magnitudes were observed in cancers of the oral cavity, pharynx, and larynx (aHR = 1.44, 1.51, and 1.62 in heavy drinkers, respectively) in our study. Results regarding the differential strength of associations between HNC subtypes and alcohol consumption have varied between studies [4,6,7,26]. A meta-analysis of 14,000 HNC patients found that the association between drinking and cancer risk was strongest in pharynx cancer; pooled OR and 95% CIs in the heavy drinking group were 5.70 (3.61–9.02), 3.93 (2.78–5.57), and 3.00 (1.76–5.11) for pharynx, oral cavity, and larynx cancer, respectively [7]. However, that study was based purely on case-control results, so recall bias might have led to overestimated associations between drinking and HNC risk. On the other hand, a prospective case-cohort study observed that oral cavity cancer was most strongly related to alcohol consumption, followed by pharynx cancer and larynx cancer (RR = 6.39, 3.52, and 1.54 in subjects drinking ≥ 30 g ethanol/day, respectively) [26]. However, that study included a limited number of HNC cases (395 overall HNC cases), far fewer than that of our study, potentially lowering their statistical power. Therefore, our results, based on a large, population-based cohort, may be much more reliable than previous results. However, unlike our study, both of the previous studies reported that larynx cancer was least associated with alcohol consumption, which the authors hypothesized might be the result of the larynx having less direct contact with alcohol than the oral cavity or pharynx [7,26]. One possible explanation for this observed difference is ethnicity; the Korean population tends to show a lower HNC incidence rate when compared with non-Hispanic white or non-Hispanic black populations, and the prevalence of each HNC subtype is also different among these populations [36]. Considering that most of the previous studies were based on Caucasian populations [7,26], ethnic differences might have resulted in weaker associations between HNC and drinking, and masked differences between HNC subtypes.

The risk of salivary gland cancer showed no definite association with alcohol consumption in our study. Because of its rarity [1], little is known about the risk factors of salivary gland cancer. However, most of the previous investigations on drinking and salivary gland cancer have found no significant association [37,38,39,40]. One population-based, case-control study which included 199 salivary gland tumor cases reported that heavy drinking was associated with increased risk in men (OR = 2.5, 95% CI = 1.1–5.7), but not in women [41]. One possible reason for this observed difference between salivary gland cancer and other HNC subtypes might be that, unlike the tissues of other HNC subtypes, the salivary glands have no direct contact with alcohol. Another possible explanation is that differences in histological type might have resulted in different relationships to alcohol; while squamous cell carcinoma is the most common pathological type in other HNC subtypes, in salivary gland cancer, adenoid cystic carcinoma and mucoepidermoid carcinoma are most common [42,43]. Further research with a larger number of cases is necessary to elucidate this relationship.

The most intriguing finding of our study was that drinking frequency is a more important risk factor of HNC than the daily amount of alcohol consumption. When stratified by average alcohol consumption, drinking frequency, but not daily amount, showed a linear relationship with HNC risk in moderate and heavy drinkers. The underlying mechanism for this finding remains unclear, but one possible explanation is that repetitive contact between a carcinogen (salivary AA) and the mucosa of the upper aerodigestive tract might lead these cells to increasingly undergo malignant transformations [44,45,46,47]. The direct carcinogenic or mutagenic effects of AA in saliva are usually observed at concentrations of higher than 40–50 μM, which can be easily reached by drinking 0.5 g of pure alcohol per kilogram of body weight [45,48]. Hence, the frequent drinking of social amount could result in prolonged exposure to the direct carcinogenic activity of AA when compared to drinking larger amounts over fewer instances. Several in vitro studies have reported that chronic, repetitive exposure to carcinogens might be required for the malignant transformation of target cells [46,47,49]. In a study using topical benzoapyrene (BAP), removal of BAP-DNA adducts from target tissues occurred faster after a single application than after chronic exposure, implying that repeated exposure-repair cycles of the genome promote an increase in carcinogen-DNA adducts [46]. To induce cancer development, carcinogens might need to be present in the target tissue for many years [50].

In our study, mild and moderate drinkers who drank 5–7 days/week showed a slightly lower risk of HNC compared with subjects who drank 3–4 days/week. Considering that the average amount of alcohol per occasion would have been less than 21 g and 42 g, respectively, among these subjects, salivary AA concentration may have been insufficient, at least for some time, to promote local carcinogenic activity.

Our study has important clinical and public health implications. Recent guidelines on drinking habits for the prevention of cancer and chronic diseases mostly advise individuals to reduce their overall consumption or daily amount without mentioning drinking frequency [51,52]. However, there are many people who frequently drink a small amount of alcohol; for example, it is common in many cultures to have a couple of drinks with lunch or dinner. Such behavior may be reinforced in some individuals by the evidence of the preventive effect of moderate alcohol consumption on cardiovascular disease risk and all-cause mortality [53,54]. Nevertheless, recent meta-analysis and large-scale studies have suggested that even a moderate level of alcohol consumption might have no benefit regarding mortality risk [55,56]. As for cancer, previous research has almost consistently shown that there is no risk threshold below which alcohol drinking is not related to incidence or mortality of cancer [8,11,57]. In the same vein, our analysis among moderate drinkers has shown that frequent drinking of relatively low doses of alcohol can also increase the risk of HNC. The findings of our study imply that a targeted public health strategy with a higher focus on drinking frequency is needed for cancer prevention in individuals who drink a small amount frequently. While HNC is relatively rare, population-wide changes in health behaviors can induce a substantial reduction in cancer incidence [58].

Strengths of our study include being the only study to examine impacts of both drinking frequency and daily amount on HNC risk, specifically. Also, our study was based on a large, population-based, representative sample. A much higher number of HNC cases (*n* = 15,832) were included than in previous investigations, allowing us to perform detailed analyses according to drinking patterns and cancer subtypes.

However, there are also some limitations in our study. First, due to the retrospective nature of our study, we could not obtain information on other components of drinking habits, such as the total duration of alcohol drinking or changes in drinking habits. As we were not able to exclude former drinkers who quit drinking recently, HNC risks among non-drinkers could have been exaggerated. Information on human papillomavirus infection, which is increasingly reported to increase the risk of oropharyngeal and oral cavity cancer [3], was also lacking. Also, we could not obtain data on genetic polymorphisms of alcohol-degrading enzymes. Previous studies have reported that several types of genetic polymorphisms could alter the activity of alcohol dehydrogenase and aldehyde dehydrogenase enzymes, and some of these genotypes are associated with the increases in HNC risk [59,60,61]. Future research might need to consider the interaction between genetic factors and alcohol drinking patterns on HNC risk. Second, information on drinking frequency or daily amount might be inaccurate because it was collected by self-reporting. Subjects might have underreported these variables or miscalculated them due to within-person variation. Third, since study subjects were limited to people who participated in the health screening program, the findings might be subject to selection bias: subjects might tend to engage in healthier behaviors than the general population. Hence, incidences of unhealthy lifestyles- such as drinking and smoking- might have been lower than in the general population, resulting in a lower incidence rate of HNC. Finally, as our study was limited to the Korean population, further studies are required to confirm the relationship between drinking and HNC found from our study in other populations.

## 5. Conclusions

In conclusion, this nationwide cohort study suggests that the frequency of drinking is a stronger risk factor for the development of HNC, especially for cancers of the oral cavity, pharynx, and larynx than the daily amount. Further research is needed on cancer prevention strategies with regard to drinking patterns.

## Figures and Tables

**Table 1:** Baseline characteristics according to average weekly alcohol consumption.

	Total (*n* = 11,737,467)	Non-Drinker (0 g) (*n* = 7,010,332)	Mild Drinker (0 < x < 105 g) (*n* = 2,781,462)	Moderate Drinker (105 ≤ x < 210 g) (*n*= 1,113,038)	Heavy Drinker (≥210 g) (*n* = 832,635)	*p*-Values
	Mean ± SD, or No. (%)					
Age (years)	54.6 ± 10.4	56.4 ± 11.0	51.8 ± 9.7	51.5 ± 9.1	52.3 ± 9.5	<0.0001
Sex (male)	5,612,691 (47.8)	2,027,999 (28.9)	1,805,061 (64.9)	992,950 (89.2)	786,681 (94.5)	<0.0001
Income (lowest quartile)	2,985,640 (25.4)	1,883,641 (26.9)	671,687 (24.2)	243,933 (21.9)	186,379 (22.4)	<0.0001
Smoking status						<0.0001
Never smoker	7,674,834 (65.4)	5,810,163 (82.9)	1,413,540 (50.8)	283,125 (25.4)	168,006 (20.2)	
Ex-smoker, <20 PY	1,064,858 (9.1)	330,639 (4.7)	426,874 (15.4)	190,356 (17.1)	116,989 (14.1)	
Ex-smoker, ≥20 PY	672,441 (5.7)	235,408 (3.4)	195,990 (7.1)	122,703 (11.0)	118,340 (14.2)	
Current smoker, <20 PY	1,042,913 (8.9)	281,892 (4.0)	404,652 (14.6)	227,469 (20.4)	128,900 (15.5)	
Current smoker, ≥20 PY	1,282,421 (10.9)	352,230 (5.0)	340,406 (12.2)	289,385 (26.0)	300,400 (36.1)	
Physical activity level						<0.0001
Non-regular	9,408,663 (80.2)	5,736,859 (81.8)	2,165,900 (77.9)	859,791 (77.3)	646,113 (77.6)	
Regular	2,328,804 (19.8)	1,273,473 (18.2)	615,562 (22.1)	253,247 (22.7)	186,522 (22.4)	
Body mass index (kg/m^2^)	24.0 ± 3.0	23.9 ± 3.1	23.9 ± 2.9	24.3 ± 2.9	24.4 ± 3.0	<0.0001
Waist circumference (cm)	81.1 ± 8.5	80.0 ± 8.7	81.5 ± 8.4	84.1 ± 7.7	85.1 ± 7.8	<0.0001
Systolic blood pressure (mmHg)	124.1 ± 15.4	123.4 ± 15.7	123.5 ± 14.9	126.8 ± 14.8	128.3 ± 15.1	<0.0001
Diastolic blood pressure (mmHg)	77.0 ± 10.1	76.1 ± 10.1	77.2 ± 10.1	79.6 ± 10.0	80.5 ± 10.1	<0.0001
Fasting glucose (mg/dL)	99.9 ± 24.5	98.9 ± 24.0	99.4 ± 23.4	103.0 ± 26.5	105.8 ± 29.2	<0.0001
Total cholesterol (mg/dL)	199.2 ± 37.0	199.8 ± 37.6	198.1 ± 35.8	199.0 ± 36.1	198.4 ± 37.0	<0.0001
HDL (mg/dL)	54.8 ± 16.7	54.5 ± 17.3	55.0 ± 15.4	55.1 ± 15.5	56.1 ± 16.6	<0.0001
LDL (mg/dL)	117.4 ± 34.1	119.9 ± 34.3	116.1 ± 33.1	111.9 ± 34.2	107.7 ± 35.3	<0.0001
eGFR (ml/min/1.73 m^2^)	85.8 ± 34.5	84.6 ± 31.0	86.7 ± 38.1	88.3 ± 41.0	89.7 ± 40.4	<0.0001
Hypertension	3,975,024 (33.9)	2,406,414 (34.3)	829,585 (29.8)	404,736 (36.4)	334,289 (40.2)	<0.0001
Diabetes mellitus	1,374,937 (11.7)	832,222 (11.9)	274,386 (9.9)	141,392 (12.7)	126,937 (15.3)	<0.0001
Dyslipidemia	2,616,859 (22.3)	1,815,516 (25.9)	564,073 (20.3)	237,270 (21.3)	181,905 (21.9)	<0.0001

Abbreviations: SD, standard deviation; PY, pack-years; eGFR, estimated glomerular filtration rate; HDL, high-density lipoprotein cholesterol; LDL, low-density lipoprotein cholesterol.

**Table 2:** Risk of overall head & neck cancer according to drinking status.

	No. (%)	Event Number	Person-Years (PYs)	Incidence Rate (Per 100,000 PYs)	Model 1 HR (95% CI)	Model 2 HR (95% CI)	Model 3 HR (95% CI)
Average alcohol consumption ^a^							
Non-drinker	7,010,332 (59.7)	7503	47,804,729.9	15.7	1 (ref.)	1 (ref.)	1 (ref.)
Mild drinker	2,781,462 (23.7)	3746	19,061,909.8	19.7	**1.06 (1.02–1.11)**	1.03 (0.99–1.07)	1.03 (0.98–1.07)
Moderate drinker	1,113,038 (9.5)	2299	7,606,759.9	30.2	**1.42 (1.36–1.50)**	**1.27 (1.20–1.33)**	**1.26 (1.20–1.32)**
Heavy drinker	832,635 (7.1)	2284	5,658,733.2	40.4	**1.74 (1.65–1.83)**	**1.47 (1.39–1.55)**	**1.46 (1.38–1.53)**
*p*-trend					**<0.01**	**<0.01**	**<0.01**
Drinking frequency (days per week)							
0	7,010,332 (59.7)	7503	47,804,729.9	15.7	1 (ref.)	1 (ref.)	1 (ref.)
1	1,897,327 (16.2)	2067	13,038,365.9	15.9	0.94 (0.90–0.99)	0.92 (0.88–0.97)	0.92 (0.88–0.97)
2	1,224,552 (10.4)	1947	8,395,959.4	23.2	**1.18 (1.12–1.25)**	**1.11 (1.05–1.17)**	**1.10 (1.05–1.16)**
3	787,753 (6.7)	1675	5,376,661.1	31.2	**1.43 (1.36–1.52)**	**1.29 (1.22–1.36)**	**1.28 (1.21–1.36)**
4	272,126 (2.3)	698	1,852,052.9	37.7	**1.63 (1.50–1.76)**	**1.42 (1.31–1.54)**	**1.41 (1.30–1.53)**
5	208,885 (1.8)	575	1,415,586	40.6	**1.62 (1.48–1.76)**	**1.39 (1.27–1.52)**	**1.38 (1.26–1.50)**
6	117,079 (1.0)	420	787,973.5	53.3	**1.79 (1.62–1.98)**	**1.51 (1.37–1.67)**	**1.50 (1.36–1.66)**
7	219,413 (1.9)	947	1,460,804.3	64.8	**1.85 (1.72–1.98)**	**1.56 (1.45–1.67)**	**1.55 (1.45–1.67)**
*p*-trend					**<0.01**	**<0.01**	**<0.01**
Daily amount (standard units per occasion)							
0	7,010,332 (59.7)	7503	47,804,729.9	15.7	1 (ref.)	1 (ref.)	1 (ref.)
1–2	827,080 (7.0)	1055	5,641,265.8	18.7	0.99 (0.92–1.05)	0.98 (0.92–1.05)	0.98 (0.92–1.05)
3–4	1,155,831 (9.8)	2006	7,906,930.4	25.4	**1.22 (1.16–1.29)**	**1.15 (1.10–1.21)**	**1.15 (1.09–1.21)**
5–7	1,622,238 (13.8)	3240	11,084,760.3	29.2	**1.41 (1.35–1.48)**	**1.26 (1.20–1.32)**	**1.25 (1.19–1.31)**
8–14	923,266 (7.9)	1668	6,330,255.9	26.4	**1.43 (1.35–1.51)**	**1.25 (1.18–1.32)**	**1.24 (1.17–1.31)**
>14	198,720 (1.7)	360	1,364,190.6	26.4	**1.47 (1.32–1.64)**	**1.27 (1.14–1.41)**	**1.26 (1.13–1.40)**
*p*-trend					**<0.01**	**<0.01**	**<0.01**

^a^ Average weekly alcohol consumption level: (1) none (0 g); (2) mild (0–105 g); (3) moderate (105–210 g); (4) heavy (≥210 g). Model 1: adjusted for age and sex. Model 2: adjusted for age, sex, income, smoking status, physical activity, body mass index, and diabetes mellitus. Model 3: adjusted for age, sex, income, smoking status, physical activity, body mass index, diabetes mellitus, hypertension, and dyslipidemia (The categories for the individual covariates were those defined in Table 1). Abbreviations: HR, hazard ratio; CI, confidence interval. Bold font indicates statistical significance.

**Table 3:** Risks of head & neck cancer subtypes according to drinking status.

	Cancer Subtypes			
	Oral Cavity HR (95% CI)	Pharynx HR (95% CI)	Larynx HR (95% CI)	Salivary Gland HR (95% CI)
Event N	4275	5598	4862	1916
Average alcohol consumption ^a^				
Non-drinker	1 (ref.)	1 (ref.)	1 (ref.)	1 (ref.)
Mild drinker	1.08 (1.00–1.17)	0.98 (0.92–1.06)	1.00 (0.93–1.08)	0.99 (0.88–1.11)
Moderate drinker	**1.34 (1.21–1.49)**	**1.27 (1.17–1.38)**	**1.33 (1.22–1.45)**	**0.95 (0.80–1.12)**
Heavy drinker	**1.44 (1.29–1.60)**	**1.51 (1.39–1.64)**	**1.62 (1.49–1.76)**	**1.14 (0.96–1.36)**
Drinking frequency (days per week)				
0	1(ref.)	1 (ref.)	1 (ref.)	1 (ref.)
1	0.98 (0.89–1.08)	**0.91 (0.84–1.00)**	**0.86 (0.79–0.95)**	0.91 (0.79–1.05)
2	**1.19 (1.07–1.33)**	1.03 (0.94–1.12)	**1.13 (1.03–1.24)**	0.99 (0.85–1.17)
3	**1.28 (1.14–1.44)**	**1.26 (1.15–1.38)**	**1.40 (1.27–1.54)**	1.10 (0.92–1.32)
4	**1.47 (1.24–1.73)**	**1.48 (1.30–1.68)**	**1.37 (1.20–1.57)**	**1.30 (1.01–1.68)**
5	**1.51 (1.26–1.80)**	**1.32 (1.14–1.53)**	**1.40 (1.21–1.61)**	0.88 (0.63–1.23)
6	**1.56 (1.26–1.92)**	**1.57 (1.33–1.84)**	**1.71 (1.48–1.99)**	0.79 (0.51–1.24)
7	**1.53 (1.31–1.78)**	**1.71 (1.53–1.91)**	**1.61 (1.44–1.79)**	**1.29 (1.00–1.67)**
Daily amount (standard units per occasion)				
0	1 (ref.)	1 (ref.)	1 (ref.)	1 (ref.)
1–2	1.02 (0.89–1.15)	0.96 (0.86–1.07)	0.95 (0.85–1.07)	1.00 (0.83–1.20)
3–4	**1.30 (1.18–1.45)**	**1.10 (1.01–1.20)**	1.09 (0.99–1.19)	1.04 (0.89–1.22)
5–7	**1.19 (1.08–1.31)**	**1.26 (1.17–1.35)**	**1.39 (1.29–1.50)**	1.00 (0.86–1.15)
8–14	**1.35 (1.20–1.52)**	**1.28 (1.17–1.41)**	**1.37 (1.24–1.51)**	0.93 (0.77–1.13)
>14	1.10 (0.86–1.40)	**1.27 (1.06–1.51)**	**1.51 (1.27–1.80)**	1.13 (0.81–1.58)

^a^ Average weekly alcohol consumption level: (1) none (0 g); (2) mild (0–105 g); (3) moderate (105–210 g); (4) heavy (≥210 g). Hazard ratios are adjusted for age, sex, income, smoking status, physical activity, body mass index, diabetes mellitus, hypertension, and dyslipidemia. Abbreviations: HR, hazard ratio; CI, confidence interval. Bold font indicates statistical significance.

## Data Availability

The data that support the findings of this study are available from the NHIS. Restrictions apply to the availability of these data, which were used under license for this study. Data are available from the authors with the permission of the NHIS.

## Supplementary Materials

The following are available online at https://www.mdpi.com/article/10.3390/ijerph182111204/s1, Table S1: Risk of head & neck cancer stratified by average alcohol consumption: Impact of drinking frequency versus daily amount, Table S2: Risks of head & neck cancer subtypes stratified by average alcohol consumption: Impact of drinking frequency versus daily amount.
