# Supplementary material for: Alcohol Drinking Pattern and Risk of Head and Neck Cancer: A Nationwide Cohort Study
Source: Int J Environ Res Public Health. 2021 Oct 25;18(21):11204. doi: 10.3390/ijerph182111204 (PMC8582646; doi:10.3390/ijerph182111204)
Supplement: Supplementary file 1 [file ijerph-18-11204-s001.zip › Supplementary table S1.pdf]

Supplementary Table S1. Risk of head & neck cancer stratified by average alcohol consumption: Impact of drinking frequency versus daily amount.

		No.	Event Number	Person-years (PYs)	Incidence Rate (per 1000 PYs)	Model 1 HR (95% CI)	Model 2 HR (95% CI)
**Drinking frequency (days per week)**							
Non-drinker	0	7,010,332	7,503	47,804,729.9	0.16	1 (ref.)	1 (ref.)
Mild drinker	1-2	2,478,062	3,007	17,009,926.3	0.18	1.01 (0.96-1.05)	0.98 (0.94-1.02)
	3-4	262,156	628	1,778,467.8	0.35	**1.37 (1.27-1.49)**	**1.29 (1.18-1.40)**
	5-7	41,244	111	273,515.8	0.41	1.17 (0.97-1.41)	1.10 (0.91-1.33)
Moderate drinker	1-2	547,950	867	3,764,429.3	0.23	**1.24 (1.15-1.33)**	**1.13 (1.05-1.21)**
	3-4	438,236	1,022	2,992,517.7	0.34	**1.56 (1.46-1.67)**	**1.37 (1.28-1.47)**
	5-7	126,852	410	849,812.9	0.48	**1.49 (1.35-1.65)**	**1.32 (1.19-1.46)**
Heavy drinker	1-2	95,867	140	659,969.6	0.21	**1.19 (1.01-1.41)**	1.07 (0.90-1.27)
	3-4	359,487	723	2,457,728.6	0.29	**1.52 (1.41-1.65)**	**1.32 (1.22-1.43)**
	5-7	377,281	1,421	2,541,035.0	0.56	**1.95 (1.83-2.06)**	**1.60 (1.51-1.70)**
**Daily amount (standard units per occasion)**							
Non-drinker	0	7,010,332	7,503	47,804,729.9	0.16	1 (ref.)	1 (ref.)
Mild drinker	1-2	807,489	992	5,512,028.7	0.18	0.97 (0.91-1.04)	0.97 (0.91-1.04)
	3-4	1,033,278	1,580	7,084,294.9	0.22	**1.15 (1.09-1.22)**	**1.10 (1.04-1.17)**
	5-7	740,175	960	5,082,401.4	0.19	1.06 (0.99-1.14)	1.00 (0.93-1.07)
	≥ 8	200,520	214	1,383,184.8	0.15	0.94 (0.82-1.08)	0.88 (0.77-1.01)
Moderate drinker	1-2	19,591	63	129,237.1	0.49	1.27 (0.99-1.62)	1.16 (0.91-1.49)
	3-4	104,558	350	702,712.6	0.50	**1.55 (1.39-1.72)**	**1.36 (1.22-1.52)**
	5-7	623,044	1,335	4,256,197.7	0.31	**1.48 (1.39-1.58)**	**1.31 (1.23-1.39)**
	≥ 8	365,845	551	2,518,612.5	0.22	**1.25 (1.14-1.37)**	**1.13 (1.03-1.23)**
Heavy drinker	3-4	17,995	76	119,922.9	0.63	**1.66 (1.33-2.08)**	**1.42 (1.13-1.78)**
	5-7	259,019	945	1,746,161.2	0.54	**1.88 (1.75-2.01)**	**1.55 (1.45-1.67)**
	≥ 8	555,621	1,263	3,792,649.2	0.33	**1.65 (1.55-1.75)**	**1.41 (1.32-1.50)**

Model 1: adjusted for age and sex

Model 2: adjusted for age, sex, income, smoking status, physical activity, body mass index, and diabetes mellitus

Abbreviations: HR, hazard ratio; CI, confidence interval
