# Supplementary material for: Alcohol Drinking Pattern and Risk of Head and Neck Cancer: A Nationwide Cohort Study
Source: Int J Environ Res Public Health. 2021 Oct 25;18(21):11204. doi: 10.3390/ijerph182111204 (PMC8582646; doi:10.3390/ijerph182111204)
Supplement: Supplementary file 1 [file ijerph-18-11204-s001.zip › Supplementary table S2.pdf]

Supplementary Table S2. Risks of head & neck cancer subtypes stratified by average alcohol consumption: Impact of drinking frequency versus daily amount.

		Cancer Subtype			
		**Oral Cavity**	**Pharynx**	**Larynx**	**Salivary Gland**
**Drinking frequency (days per week)**					
Non-drinker	0	1 (ref.)	1 (ref.)	1 (ref.)	1 (ref.)
Mild drinker	1-2	1.02 (0.94-1.12)	0.95 (0.89-1.03)	0.95 (0.87-1.03)	0.94 (0.83-1.07)
	3-4	**1.39 (1.18-1.64)**	**1.17 (1.02-1.35)**	**1.26 (1.10-1.45)**	**1.31 (1.02-1.69)**
	5-7	1.37 (0.97-1.95)	0.89 (0.63-1.26)	1.01 (0.75-1.38)	1.19 (0.65-2.15)
Moderate drinker	1-2	**1.29 (1.11-1.49)**	1.08 (0.96-1.22)	**1.17 (1.03-1.33)**	0.90 (0.71-1.15)
	3-4	**1.39 (1.21-1.61)**	**1.41 (1.26-1.57)**	**1.49 (1.33-1.67)**	1.04 (0.82-1.31)
	5-7	**1.38 (1.12-1.70)**	**1.38 (1.17-1.62)**	**1.32 (1.13-1.55)**	0.85 (0.57-1.28)
Heavy drinker	1-2	1.22 (0.86-1.68)	0.79 (0.58-1.08)	1.23 (0.92-1.63)	1.29 (0.82-2.01)
	3-4	**1.23 (1.04-1.47)**	**1.38 (1.21-1.56)**	**1.48 (1.29-1.69)**	1.19 (0.93-1.53)
	5-7	**1.62 (1.42-1.84)**	**1.70 (1.54-1.87)**	**1.74 (1.58-1.91)**	1.09 (0.87-1.37)
**Daily amount (standard units per occasion)**					
Non-drinker	0	1(ref.)	1(ref.)	1(ref.)	1(ref.)
Mild drinker	1-2	1.01 (0.89-1.15)	0.92 (0.82-1.03)	0.94 (0.83-1.06)	0.97 (0.81-1.17)
	3-4	**1.24 (1.11-1.38)**	1.04 (0.95-1.14)	1.03 (0.93-1.14)	1.05 (0.89-1.24)
	5-7	0.95 (0.82-1.10)	1.02 (0.91-1.14)	1.07 (0.95-1.21)	0.87 (0.71-1.08)
	≥ 8	0.92 (0.70-1.22)	0.80 (0.64-1.01)	0.88 (0.68-1.13)	1.10 (0.77-1.56)
Moderate drinker	1-2	0.98 (0.55-1.72)	**1.57 (1.10-2.23)**	1.10 (0.75-1.62)	1.71 (0.85-3.44)
	3-4	**1.57 (1.26-1.94)**	**1.35 (1.13-1.61)**	**1.36 (1.15-1.62)**	0.99 (0.65-1.50)
	5-7	**1.32 (1.16-1.50)**	**1.29 (1.16-1.42)**	**1.47 (1.33-1.62)**	1.02 (0.83-1.25)
	≥ 8	**1.32 (1.11-1.57)**	**1.17 (1.01-1.35)**	1.09 (0.93-1.28)	**0.71 (0.52-0.98)**
Heavy drinker	3-4	**2.07 (1.38-3.11)**	**1.52 (1.06-2.18)**	1.15 (0.78-1.68)	1.35 (0.61-3.03)
	5-7	**1.41 (1.20-1.65)**	**1.59 (1.42-1.78)**	**1.66 (1.49-1.86)**	1.22 (0.95-1.58)
	≥ 8	**1.43 (1.25-1.63)**	**1.46 (1.32-1.62)**	**1.65 (1.48-1.83)**	1.09 (0.88-1.34)

Hazard ratios are adjusted for age, sex, income, smoking status, physical activity, body mass index, and diabetes mellitus.
